# Dread-Bound News

**DOI:** 10.3201/eid0802.AD0802

**Published:** 2002-02

**Authors:** Zoe Haugo

**Affiliations:** Atlanta, Georgia

**Keywords:** antimicrobial resistance, Taiwan

It is difficult

to get the news from poems

yet men die miserably every day

for lack

of what is found there.

“Asphodel, That Greeny Flower” by William Carlos Williams, a physician and poet

We cower under stars and stripes
While horrors unfold and threaten the future
Armies, governments, news hounds
Chase their tails and the tales of violence,
But the crucial culprit, collective fear,
Pervades our lives.
Fear that infectious disease of humans,
With strains varied as the common cold,
Has no vaccine or cure and lurks disguised,
Evades diagnosis.
In fear we cannot, must not hide
From the public info-network
That slams us with a headline epidemic
Prompting us to wade in the bay of panic.
The enemy’s friend and foe,
It gives voice to the great unknown,
Paints faces on the monster
Who hides under our beds.
United, we wait for disaster
Fearing to suck our thumbs,
Lest mama's apron strings be dusted with anthrax.
A germ becomes a giant!

